# The pandemic’s unseen wounds: COVID-19’s profound effects on mental health

**DOI:** 10.1097/MS9.0000000000001223

**Published:** 2023-09-01

**Authors:** Rishabh Chaudhary, Manni Rohilla, Samrat Chauhan, Monika Saini, Shahbaz Aman, Hrithik Singla, Ayesha bibi, Sohel Ahmed, Shalini Shriwastav, Narinder Kaur, Jai Dev, Rishabh Chalotra, Thakur Gurjeet Singh, Sachin Mehta

**Affiliations:** a M.M. College of Pharmacy; b Department of Microbiology, M.M. Institute of Medical Science and Research, Maharishi Markandeshwar (Deemed to be University), Mullana, Ambala, Haryana; c Chitkara College of Pharmacy, Chitkara University; d Swami Vivekanand College of Pharmacy, Rajpura, Punjab; e Department of Pharmacology, Central University of Punjab, Bathinda, India; f Birat Medical college Teaching Hospital, Kathmandu University, Nepal

**Keywords:** anxiety, COVID-19, depression, direct population, indirect population, mental health

## Abstract

**Objective:**

This review aims to explore the impact of the COVID-19 pandemic on mental health, with a focus on the physiological and psychological consequences, including comorbidities. The goal is to understand the direct and indirect populations affected by mental distress and identify potential interventions.

**Methodology:**

A comprehensive literature search was conducted using various databases, including Google Scholar, ResearchGate, ScienceDirect, PubMed, PLoS One, and Web of Science. The search utilized relevant keywords to investigate the direct and indirect impacts of COVID-19 on mental health. The selected articles were critically evaluated and analyzed to identify key findings and insights.

**Main findings:**

Mental health, being an intrinsic component of overall well-being, plays a vital role in physiological functioning. The COVID-19 pandemic, caused by the emergence of the novel SARS-CoV-2 virus, has had a devastating global impact. Beyond the respiratory symptoms, individuals recovering from COVID-19 commonly experience additional ailments, such as arrhythmia, depression, anxiety, and fatigue. Healthcare professionals on the frontlines face an elevated risk of mental illness. However, it is crucial to recognize that the general population also grapples with comparable levels of mental distress.

**Conclusion:**

The COVID-19 pandemic has underscored the significance of addressing mental health concerns. Various strategies can help mitigate the impact, including counselling, fostering open lines of communication, providing mental support, ensuring comprehensive patient care, and administering appropriate medications. In severe cases, treatment may involve the supplementation of essential vitamins and antidepressant therapy. By understanding the direct and indirect impacts of COVID-19 on mental health, healthcare providers and policymakers can develop targeted interventions to support individuals and communities affected by the pandemic. Continued research and collaborative efforts are essential to address this pervasive issue effectively.

## Introduction

Highlights

- COVID-19 has had a devastating impact globally, causing physical and mental health issues.
- After recovering from COVID-19, individuals may experience comorbidities such as arrhythmia, depression, or anxiety.
- Front-line healthcare workers and their families are severely affected by the fear of infection which significantly affected their mental health.
- Psychological support is crucial for sustaining healthcare services and providing proper treatment.
- Support programs, counselling, and motivation can help reduce stress levels and fear.

Mental health is an integral part of every stage of life from adolescence to adulthood and represents a state of emotional, psychological, and social well-being. It has become one of the most neglected aspects of society—mental health problems arising from various biological factors, life experiences, and family history of mental health. Mental illness includes anxiety, behavioural changes, mood swings, and disturbances in sleeping patterns^1^. In December 2019, a series of acute and atypical respiratory diseases was identified in Wuhan, China. The source of the illnesses was attributed to a novel coronavirus, named the SARS-CoV-2, and the subsequent disease it caused was named the COVID-19, and on 11 March 2020 it was declared a pandemic by the WHO^2,3^.

Mental health is a broad term that can be categorized based on normal, mild, moderate, severe, and extremely severe. In search of literature, various populations, like direct or indirect, show different types of symptoms that are directly related to the mental behaviour of the individual. Based on these symptoms, we have established the categorized behaviour as shown in the previous line. Normal mental health is considered normal when populations are working normally in the community or in hospitals and do not face any mental burdens like stress, depression, irritation, excessive fear, loss of confidence, hand-tingling behaviour, etc.^1^, . Normal populations show symptoms like positive mood, ability to cope with everyday challenges, good concentration and focus, adequate sleep and appetite, and ability to maintain healthy relationships. Mild mental health symptoms like occasional mood swings, mild anxiety or worry, decreased motivation or interest in activities, mild difficulty concentrating, slight changes in sleep or appetite, etc., are considered mild symptoms in this study. Whereas moderate symptoms like frequent mood swings, increased anxiety or worry, a noticeable decrease in motivation or interest, difficulty concentrating, disturbed sleep patterns, a change in appetite, etc., are considered moderate symptoms for this study. Another one like severe symptoms include intense and persistent mood swings, overwhelming anxiety or panic, loss of interest in previously enjoyed activities, impaired cognition and memory, severe sleep disturbance, and significant changes in appetite, etc. These are the symptoms of severe mental health conditions^4^. Extremely severe symptoms include substantial interference with daily functioning, extreme and uncontrollable mood swings, severe anxiety or panic attacks, complete loss of interest in activities, severe cognitive impairment, severe sleep disorders, drastic changes in appetite, profound interference with daily functioning, etc. These all types of different classifications of symptoms are used in this study so that based on direct and indirect populations, we can easily categorize mental health by using the literature survey^5^.

The COVID-19 pandemic has severely impacted our physical, emotional, and social health and well-being, but the mental health aspects related to the disease have been neglected. Mental health represents a state of physiological, emotional, and social well-being^6^. The COVID-19 pandemic accelerates symptoms of long-term psychological stress. Individuals with COVID-19 may encounter anxiety, depression, delirium, and insomnia^7^. The COVID-19 pandemic produces a diverse mental and psychological inference index for a wide range of high-risk possibility groups, such as healthcare workers working on the front-line, COVID-19 patients, and quarantined individuals^8^. Individuals with good social support are less likely to develop anxiety and depression symptoms. Poor lifestyle behaviours and indulging in activities like (poor diet, smoking, lack of exercise, and sleep) lead to an increased prevalence of mental health disorders, which was boosted by COVID-19^4^. COVID-19 significantly affects young children (a susceptible population); the pandemic changes the lifestyle of students, and the threat of getting infected generates mental fear and depressive and anxious symptoms^9^. Anxiety and depression persist from adolescence to adulthood due to a lack of mental arbitration and an increase in the risk of age-related diseases such as cardiovascular disease^10^. Nursing care facilities that are directly involved in caring for COVID-19 patients and working in quarantine zones are more likely to be affected by psychological and mental distress^5^.

Individual strength, which refers to the person’s ability to fight anxiety, psychological distress, and depression stress, is likely to play an essential role in fighting mental symptoms^11^. Suicidal ideation among adolescents due to fear of getting infected increases the need for consultation with healthcare professionals due to the emerging stress surrounding this pandemic^12^. Both psychological and mental stress negatively impact both the mother and baby during pregnancy, and COVID-19 elevates anxiety symptoms in pregnant individuals. This anxiety significantly affects foetal development and may interfere with sleep and nutrition, thereby affecting maternal mood^13^. It also increases loneliness during a pandemic and the fear of becoming infected by the necessity of consultation with a healthcare professional^14^.

Patients with COVID-19 and other comorbidities can exhibit various signs and symptoms. The most common symptoms are fever, headache, nausea, difficulty breathing, and headache^15^. However, in most cases, a patient’s condition can worsen and damage the respiratory system. In co-morbid conditions such as depression, the patient’s condition can become fatal and lead to death. The less common symptoms are sores that threaten patients, aches, pain in the body, rashes on the skin, and diarrhoea, affecting the patient’s health^16^. Various pharmacological and non-pharmacological treatments are available, and all have therapeutic potential against the virus. Multiple types of vaccinations are available on the market. However, they are not very effective, and several clinical and sub-clinical studies are currently being undertaken to find a gold-standard therapeutic regimen with high efficacy and few side effects^17^. There is a great need for attention to patients’ mental health during COVID-19 to provide them with safe treatment and interventions to improve mental distress and decrease the mental stress emerging from COVID-19^18^. According to scientists, the ratio of co-morbid conditions increases daily among patients. This co-morbid ratio directly affects doctors, nurses, and healthcare workers and indirectly affects parents, schoolteachers, the government, and private workers^19^. As we know, the ratio of co-morbid diseases increases, so COVID-19 is spread all over the population, from patients to doctors and then to our family members (including children), which are directly affected and, in other cases, indirectly affected, such as from government and private workers to our family members. There is an increase in pharmacological side effects of COVID-19, which requires immediate measures to lower the level of depression and psychological disorders^20^.

## Impact of COVID-19 on mental health—direct population

Mental health refers to a state of emotional, psychological, and social well-being. It determines one’s ability to cope with the stresses of life and work activities, and the ability to provide a meaningful contribution to society^21^. It has become one of the most neglected areas in public health. The recent COVID-19 outbreak has increased mental health problems in communities^22^. COVID-19directly affected the population, including front-line healthcare workers, nurses, paramedical, and supporting staff. Treating physical illness is a primary focus of healthcare workers, but good mental health also promotes speedy recovery^23^. Front-line workers are physicians, nurses, and support staff who have direct contact with patients and work 24-h day and night shifts while experiencing anxiety and depression. The primary cause of long-term psychiatric stress and anxiety among healthcare workers is a high workload and overload^24^. Adolescents living at home without companions suffer from anxiety and depression^25^. Generally, the fear of getting infected with COVID-19 is seen more in females than in males and people with no chronic illness^26^. COVID-19 significantly affects the maternal mental health of pregnant women, leading to the development of anxiety and depression and fear of child abnormality and miscarriages^27^. Families associated with them also experience fear anxiety, mental stress, and contamination. Various service providers and support systems can help to reduce stress levels. Greater attention, appropriate intervention, and social support may aid in stress reduction^28^.

### Impact of COVID-19 on doctors

The COVID-19 outbreak leads to the rapid development of COVID-19 health centres and the conversion of hospitals to quarantine centres, forming a rapid response team of doctors and physicians to cope with stress^29^. Therefore, fear of becoming infected and fear of transmission of viruses to their families develop symptoms of anxiety and depression and increase their chances of developing long-term psychological disorders. Doctors have contributed significantly to patient care by working in various wards, emergency rooms, intensive care units, etc. Physicians are directly involved in treating COVID-19-infected patients^30^. Therefore, the fear of becoming infected and transmitting viruses to their families leads to anxiety and depression. Due to long-term isolation from friends and family, irregular working patterns, and sleep disturbances, stress sets in doctors^31^. Doctors must be provided with adequately scheduled working shifts and in other areas^32^. Appropriate guidelines on handling emergency cases and treating infected patients, as well as guidelines for the proper use of personal protection kits and sanitization facilities, must be provided to ensure the safety of physicians and help relieve their stress. Long working hours and excessive shifts contribute to increased depression in physicians. Doctors must receive appropriate intervention and professional assistance^33^. Family members of healthcare workers experience extreme anxiety, depression, and apprehension about infecting their loved ones. A survey in Wuhan, including 1257 Chinese front-line healthcare workers, showed prevalence rates of 50.4% for depression, 44.6% for anxiety, and 71.5% for distress^34,35^. Personal factors, including sleep disturbances, separation from the family, and fear of getting infected, contribute to depression. Heavy workloads for excessive time and sometimes a lack of protection kits develop fear. To better understand the psychological needs of doctors and improve overall patient care, great attention, interventions, and appropriate professional help must be provided to boost the confidence of healthcare professionals^36^. Table 1 shows the percentage of doctors affected by mental health in the direct population.

**Table 1:** Direct impacts of COVID-19 on healthcare professionals-doctors

Severity	Depression (%)	Anxiety (%)	Stress (%)
Normal	71.4	78.6	66.7
Mild	12	10	5.0
Moderate	11	24	28
Severe	14	5.6	10
Extremely severe	10	5	4.5

The DASS-21 SCALE (Depression, Anxiety, Stress Scale-21) assessed depression, anxiety, and stress. The healthcare workers were divided into five categories: normal, mild, moderate, severe, and extremely severe using this scale. Healthcare professionals assisting patients with COVID-19 display much higher depression, anxiety, and stress scores than shown in Table 1. Urgent measures and interventions, including providing good social support and motivating them, can help lower depression and stress levels^36^.

### Impact of COVID-19 on nurses

Nursing is an intrinsic part of hospitals or healthcare systems that promotes health and prevents illness. Nurses perform various hospital functions ranging from gathering patient information to ensuring appropriate patient care treatment^37^. Due to the COVID-19 pandemic, nurses have experienced increased workload and pressure, which has led to depression and anxiety due to long working hours^28^. It has been observed that a large percentage of nurses’ experience anxiety and are at increased risk to occupy posttraumatic stress disorder and depression. According to recent studies, it has been observed that females working in hospitals experience and tend to be significantly affected by sudden stress compared to males^38^. Nurses contribute to the primary working staff in hospitals and are directly involved in patient care, assisting with medical supplies, and caring for patients. Therefore, these individuals tend to develop anxiety and long-term stress disorders more rapidly. Nurses are generally involved in caring for infected patients and spending a lot of time with them, which leads to a higher risk of developing mental stress disorders^39^. Families associated with nursing staff also suffer from long-term isolation from their loved ones and experience long-term stress disorders and fear of transmitting viruses to their loved ones. Studies have reported that mental stress disorders are observed more frequently in nurses than in physicians or other staff during the handling of infected patients^40^. The quality of nursing care facilities depends on the quality of the environment, which can be achieved by motivating nurses and improving their health by providing appropriate interventions. Providing good social support and helping them with educational aids and therapies can help them cope with increased workload and pressure and lower their depression, anxiety, and stress levels^41^. Table 2 shows the percentage of nurses affected by mental health issues in the direct population.

**Table 2:** Impacts of COVID-19 among nurses

Severity	Depression (%)	Anxiety (%)	Stress (%)
Normal	60.5	50	74.3
Mild	19.7	7.9	9.9
Moderate	9.9	20.4	9.9
Severe	5.3	9.9	2.6
Extremely severe	4.6	11.8	3.3

A study was conducted in Nepal to evaluate nurses’ mental status. A total of 152 nurses were taken into account assessed depression, anxiety, and stress levels. The mean age group of 24.09 years was studied. Table 2 depicts the results of studies using the DASS (Depression, Anxiety, and Stress Scale)^42^. Table 2 illustrates that (9.9%) of the nurses showed severe and extremely severe levels of depression. (20.4%) of nurses showed moderate to severe anxiety levels. Severe and extremely severe levels were observed in 3.3% of the nurses. Therefore, it was concluded that depression, anxiety, and stress levels were observed among nurses during the pandemic. Appropriate interventions and boosting confidence levels can help improve nurses’ mental health. Therefore, great care must be taken to ensure the mental health of nursing care staff working in hospitals to improve overall patient care and reduce psychological stress disorders^43^.

### Impact of COVID-19 on supporting and paramedical staff

Coronaviruses have caused turbulence in the healthcare sector and society. During this outbreak, there was immense pressure and workload on front-line supporting and paramedical staff working in hospitals and COVID-19 quarantine centres^44^. The rapid development of quarantine centres is essential for medical staff to handle crises. Front-line paramedical and supporting teams suffer from massive and intensive working hours^45^. Work overload causes depression, anxiety, and stress symptoms, and bears more chances of being infected by COVID-19. Long working hours lead to physical burnout, which increases symptoms of depression, anxiety, and stress. Stress increases the chance of developing long-term psychiatric disorders and makes individuals more vulnerable to mental disorders. Paramedical and supporting staff contribute by performing appropriate diagnostic procedures, taking patients’ medical histories, assisting supplies of medicines, and taking blood samples for testing and diagnostic procedures^46^. Associated families with supporting and paramedical staff also suffer from severe stress and depression from getting infected with their loved ones. Increasing deaths due to COVID-19 develop symptoms of fear among workers and generated an alarming need to cope with their stress to provide sustainable services. The distribution of appropriate personal protection kits, equipment, and intervention guidelines for managing stress must be mentioned. Sanitation and individual measures must be strictly followed to prevent infections^47^. The supporting staff showed symptoms of feeling condemned and ignored during the pandemic surge, and an excessive workload elevates their stress. The great need for attention must be paid to them, and the provision of rest areas and maintenance of working hours can help relieve their stress. Appropriate counselling sessions and consultation with health professionals can help them counter their stress and boost their morale, which can counteract their stress and anxiety^48^.

Table 3 shows the percentage of support and paramedical staff affected by the COVID-19 pandemic. Workers were divided into five levels of severity: normal, mild, moderate, severe, and extremely severe. Workers on the front-line scored much higher. The results presented present an alarming need to understand the psychological needs of workers during the pandemic and provide them with appropriate interventions and counselling sessions to help them cope with their stress.

**Table 3:** Direct impact on supporting and paramedical staff

Severity	Depression (%)	Anxiety (%)	Stress (%)
Normal	73	78.9	68.7
Mild	11.5	12.3	8
Moderate	10	22.5	27.5
Severe	12.5	5.9	8.9
Extremely severe	8.8	4.7	4.2

### Impact of COVID-19 on mental health—indirect population

Mental health refers to cognitive, behavioural, and emotional well-being. The co-morbid conditions associated with COVID-19 include depression, anxiety, and stress^49^. The COVID-19 outbreak has been found to indirectly affect the mental health of society, youth, and children. The population includes the society, youth, children, pregnant women, and young adolescents. Society and youth generally suffer from the fear of being infected by the virus, which causes anxiety, depression, and stress^50^. The abrupt change in lifestyle caused by working from their offices and institutions to their homes impacts mental health. Specific working hours and home office schemes have generally been adopted to cope with the virus. Long-term social isolation causes anxiety disorders and contributes to depression and stress disorders. Overthinking and negative emotions frequently dominate and spread fear, leaving the community vulnerable to stress^51^ also seems to indirectly affect research and scientific fields. Clinical studies were postponed and terminated because of the pandemic, which affected the quality of the research. Several clinical trials have been delayed because of fear of viral infection. According to a previous study^52^. The fear of becoming infected and transmitting the virus to their children affects their mental health and generates long-term psychological stress. Individuals with prepsychiatric disorders were affected more significantly. Societies suffer from economic limitations and difficulties accelerated by COVID-19, resulting in long-term depression, anxiety, and stress disorders. Lack of social isolation results in stress development. Urgent measures and appropriate interventions are required to provide complete psychological support to children and society in order to motivate them and overcome their fear^53^.

### Impact of COVID-19 among youth and children

Youths and children represent the country’s future and will lead the country. These are the building blocks of the nation. The good mental health of youth and children provides a gateway for the country’s future. Using their energies in a right and constructive manner can lead to the development of the country^54^. The recent spread of coronavirus has led to harmful consequences for mental health. A sudden change in lifestyle from attending lectures at colleges and institutions to online classes increases the risk of developing long-term mental disorders, and a rapid surge in anxiety, depression, and stress has been observed^55^. Due to a lack of social interaction and social isolation, children show anxiety and stress-related symptoms that contribute to the development of psychological stress. In general, social isolation promotes feelings of loneliness and suicidal ideation. Youths and children who interact and have contact with COVID-19-infected patients have a greater possibility of developing stress disorders^56^. Increased fear of being infected by viruses and social isolation have terrible consequences on mental health. COVID-19 generates a sense of fear among youth and children, which can trigger signs of emotional stress, sickness, and severe cases, including death, arising from real-time responses to threats^57^. The development of these symptoms results in poor adaptation to daily life and work activities, and a depressed state of mind. Children with previous physical illnesses have suffered badly from the pandemic. Spreading positivity and engaging in activities to distract them from stress and motivating and guiding them help relieve their anxiety symptoms and reduce mental health symptoms^58^. Healthcare psychology providers and decision makers should develop measures to eliminate the future risk of developing emerging mental illnesses^59^. Table 4 represent the indirect impacts of COVID-19 on youth and children.

**Table 4:** Impact of COVID-19 among youth and children

Severity	Depression (%)	Anxiety (%)	Stress (%)
Normal	53	65	52
Mild	20	24	23.5
Moderate	18.4	18	17.5
Severe	8	9.7	10.5
Extremely severe	6.7	7.2	7.5

Table 4 depicts the prevalence rates of depression, anxiety, and stress among the youth and children. The results were evaluated using online surveys and different scales, which included (DASS-21), (PHQ-9), and (GAD-7). The recent spread of coronavirus has resulted in high prevalence rates of post-psychological disorders among youth and children. The pandemic has resulted in a lack of social interaction and elevated the symptoms of loneliness and suicidal thoughts. Greater attention is needed, and extreme measures must be taken to reduce stress. Counselling sessions and meetings with healthcare professionals can help reduce stress and mental stress levels.

### Impact of COVID-19 on pregnant women

Pregnancy is a crucial time for both the mother and the baby. Stress, anxiety, or depression can have negative consequences for the foetus and mother. Studies have shown that psychological stress is more greatly affected by women than by men^60^. Due to the pandemic, women suffer from increased prenatal anxiety and depression, which can affect the health of the mother and foetus. According to previous studies, the increased anxiety experienced by the mother as a result of the pandemic increases the risk of miscarriage and congenital disabilities, such as low foetal weight, premature delivery, and growth defects^61^. Behavioural changes and changes in the brain structure of infants have also been observed. Pregnant women with a previous history of depression tend to have a broadly accelerated risk of depression and anxiety, which leads to poor physical health of the mother and baby. A study conducted in Turkey by ER *et al*.^62^ showed an increase in psychological stress among pregnant women. Families also suffer from anxiety due to the fear of transmission of viruses to their children. Pregnant women also suffer from increased anxiety about not getting enough attention and care due to increased COVID-19 cases, resulting in stress.

It has been observed that about 10–15% of pregnant women suffer from anxiety and depression, which can affect their health and the mother’s^63^. Pregnancy generates different roles and duties for the mothers. Providing adequate social support can help fight stress and depression. Emotional and behavioural changes can occur in women due to increased stress. It is possible to overcome stress by providing women with appropriate interventions, emotional support, and by building up their morale. According to previous studies, sensitive social and psychological relationships help one to bring positive thoughts and boost confidence, resulting in a lower level of depression, anxiety, and stress^64^. Prenatal stress can be countered by maintaining good care of patients with social support and physical activity, which can reduce depression and anxiety. Appropriate interventions can reduce the risk to newborns during the pandemic after the detection of anxiety and depressive symptoms are detected^18^. As per a survey in Iran, pregnant women visiting Tabriz health centres were evaluated for depression, anxiety, and stress. A total of 205 pregnant women were assessed and the results are presented in Table 5.

**Table 5:** Indirect impacts on pregnant women

Severity	Depression	Anxiety	Stress
Normal	67.3	56.1	68
Mild	12.7	17.6	7.8
Moderate	10.7	12.2	15.6
Severe	7.3	6.3	7.8
Extremely severe	6	8	7.2

It was concluded that (67.3%) of women were normal and (32.7%) of women experienced depression. In addition, (32.7%) of women showed symptoms of stress. (56.1%) of women showed a normal response in an anxiety test, while the remaining (43.9%) had anxiety symptoms^65^. These findings indicate an increased risk of depression and anxiety, and significant psychological changes in women during the pandemic, with short-term and long-term consequences for the health of the foetus and mother. Increased fear among pregnant women visiting healthcare centres has been observed during the pandemic. Providing good social support and support systems during this vulnerable period can help them cope with and handle psychological stress^66^.

### Impact of COVID-19 among researchers

The COVID-19 pandemic has severely disrupted lives worldwide and brutally impacted clinical research and the scientific community. During the pandemic, there was a rapid increase in the number of research and scientific papers related to the COVID-19 pandemic peaked, while other diseases were neglected^67^. The COVID-19 pandemic leads to the suspension of non-COVID research, which has dramatically affected the scientific community. Various preventive measures, such as suspension from their labs and working from their homes, affect the quality of ongoing research. The pandemic has reduced the number of clinical trial work subjects and databases. Long-term research activities, including living organisms, have been disrupted entirely, severely impacting researchers and the scientific community. The pandemic has resulted in many clinical trials being either postponed or abandoned^68^. The lack of research infrastructure and the inability to access labs significantly affect the quality of ongoing research. COVID-19 produces both short-term and long-term health effects on people and affects healthcare services such as medical research. Closing economics during a pandemic is also a major reason for closing down labs for non-COVID-19-related research, which leads to low research training activities and funding^69^. Most clinical studies have been suspended, except those evaluating life-saving medicines, and most ongoing clinical trials have been closed or postponed. Clinical trials are being conducted through home monitoring systems with the help of virtual aids to lower the candidate’s risk of COVID-19 infection and keep them away from infection risk. Public health centres remain closed because of social distancing norms that support them from conducting lab experiments. According to these studies, COVID-19-related research papers have an eight-fold drop in output, while non-COVID-19-related reports have an 18–19% drop.

On average, clinical trials received a drop of ~28% in the research area. Most non-COVID clinical research has been halted due to social distancing norms and measures, resulting in a decrease in study subject enrolment and a delay in data input into clinical trial databases^70^. Research-related employment was severely affected by the COVID-19 pandemic, which halted the growth of young researchers pursuing their jobs or careers in the research field and displayed another aspect of the pandemic^71^. It is necessary to use innovative strategies and a planned approach to advance this curve and continue conducting quality research and proposed clinical trials^72^. Patient safety is of utmost importance. Therefore, reopening and cleaning sites are required to continue clinical studies. This review focuses on the importance of clinical studies and highlights the importance of scientific research. The pandemic presents long-term health consequences for society and the medical research field. There is a dire need to mark and identify clinical research problems and provide appropriate aid to cope with difficulties in ongoing research^73^.

Table 6 depicts the percentage of research activity hindered before and after the suspension of work with scientists and working hours. Before the pandemic, the researcher was free to work with scientists and conduct research. The results showed that a decrease in working hours, suspension of clinical trials, and non-COVID-related research was significantly affected and disrupted during the outbreak. Greater attention and interventions must be taken to counter the stress and work loss due to the outbreak must be taken into account.

**Table 6:** Impacts of COVID-19 among researchers

Impacts	Before the pandemic (%)	After the pandemic (%)
Working hours	99	45
Work with scientist	97	10
Clinical trials	95	30
Non-COVID-related research	96	10.5

### Impact of COVID-19 on society

The COVID-19 pandemic has completely changed and altered the routine lives of people, and this sudden change has affected their mental health and generated divergent levels of stress, depression, and anxiety^74^. A pandemic creates a sense of stress and fear in society, which leads to increased symptoms of psychiatric comorbidities, such as stress, depression, and anxiety disorders. Families of COVID-19-infected patients experience extreme stress and depression and fear of becoming infected and being separated from their loved ones for an extended period^75^. Studies have shown that, in addition to the fear of a pandemic, other factors, such as a lack of social interaction, fear of being separated from their families, a sense of loneliness, and economic capabilities, are essential factors that have a significant impact on society^76^.

Generally, negative emotions dominate and people may not be able to overcome their fear efficiently. People were advised to work from their homes and were terminated from attending offices. This sudden change in lifestyle, as they work from their offices to work from home, develops stress-related symptoms^77^. Lack of social and physical interaction leads to different levels of mental health conditions such as depression, anxiety, and psychological stress. According to these studies, symptoms of depression, anxiety, and stress during the early stages of a pandemic may be a precursor for predicting different mental health levels^78^. COVID-19 results in a complete lockdown, so specific behavioural changes are generated in individuals, from fear of getting infected to insomnia, anger, alcoholism, and mental health conditions, such as depression and stress disorder. People with previous health disorders and problems suffer from fear of visiting healthcare centres. A pandemic lead to the shutting down of economies and threatens people’s jobs, resulting in the loss of employment and the generation of fear in society. People suffer from loss and fear of economic inability to cope with their daily needs and requirements^79^. Loss of working hours and employment lead to symptoms of depression and long-term psychological disorders^80^. Families of front-line healthcare workers, such as physicians, nurses, pharmacists, paramedics, and others, who work day and night, are terrified of being infected by the virus and infecting their loved ones. There is an urgent need for accurate detection of and access to individuals’ health outcomes, early intervention techniques, and plans to prevent COVID-19-related fear.

A pandemic generates a need to consult healthcare professionals to provide appropriate intervention strategies and techniques to overcome their fear. Specific mental health promotion strategies and digital interventions can help lower depression and stress levels and build self-confidence to fight stress. Healthcare professionals must monitor and promote healthy activities, such as guiding and motivating people about online psychology therapies, discussing their health benefits, and instructing people about general skills regarding handling the pandemic. Overcoming loneliness by encouraging them and counselling can decrease mental health conditions^81^. Table 7 presents the percentage of indirect impact of COVID-19 on society.

**Table 7:** Impact of COVID-19 on society

Severity	Depression (%)	Anxiety (%)	Stress (%)
Normal	59.8	60.5	69
Mild	22	17	19
Moderate	19.5	21	17.5
Severe	5.3	9.9	5
Extremely severe	4.2	9.8	4.5

Table 7 depicts the prevalence rates of depression, anxiety, and stress in the society during the pandemic. The results were evaluated using various questionnaires, online surveys, and scales, including the DASS-21, PHQ-9, and GAD-7. Society shows much higher depression, anxiety, and stress scores during the outbreak and presents an alarming need to provide appropriate intervention and support. Various counselling policies and the inclusion of healthcare professionals can help reduce stress levels.

### Impact of COVID-19 on the different geographical regions

Of the five geographical regions, Southeast Asia, North America, and South Asia were severely and immensely affected, and showed much higher prevalence rates of depression, anxiety, and stress. Middle East and Southern Europe showed lower prevalence rates during the crisis period. A pandemic causes fear in society, which generates different levels of short-term and long-term mental health disorders. As the pandemic seems to have originated and spread in Southeast Asia, it has created chaos in the southeast region. It then spreads to other geographical areas, which disrupts people’s normal lives. After the southeast region, North America and South Asia seem to have been immensely affected. A pandemic causes lockdown in the area, which leads to unemployment and fear among society to cope with their daily needs and fear of becoming infected. The respondents’ levels of stress and anxiety present an alarming and urgent need to take immediate measures and interventions to understand the various levels of psychological stress faced by society before the development of long-term psychological stress disorders^82^. Policymakers and providers must be concerned about the emerging mental healthcare illness that results in insomnia, development of suicidal thoughts, depression, anxiety, and long-term psychological stress to support the population from developing stress. Immediate psychological support and counselling sessions can help lower stress levels^83^. Understanding risk factors and having a trusted support system can help people cope with stress and reduce psychological stress. Various healthcare service providers and governmental organizations can provide psychological assistance and design appropriate therapies and educational programs to help improve the mental health status of society. Table 8 shows the total percentage of the population affected by depression, anxiety, and stress in the different geographical regions.

**Table 8:** Depression, anxiety, and stress among the different geographical regions

Geographical regions	Severity	Depression (%)	Anxiety (%)	Stress (%)
Southeast Asia	Normal	53	68	76
	Mild	38	34.5	37
	Moderate	25.5	30.5	31.5
	Severe	16	16	18
	Extremely severe	11.5	10	12
North America	Normal	79.5	74	71.2
	Mild	32	31.5	34
	Moderate	27	28	29.5
	Severe	12.5	14	11.5
	Extremely severe	9.5	11	8.5
South Asia	Normal	75	72	59
	Mild	28	32	41
	Moderate	23.5	25	28
	Severe	13.5	13.5	15
	Extremely severe	10	9	8.1
Middle East	Normal	47	63	59
	Mild	16	8	14
	Moderate	22	14	16
	Severe	12	12	13.5
	Extremely severe	11.5	9.5	8
Southern-Central Europe	Normal	72	69.5	70
	Mild	27	28.5	22.5
	Moderate	24	19	22
	Severe	20.5	22.5	21.8
	Extremely severe	9	11	14

Table 8 Depicts the Prevalence of depression, anxiety, and stress in East Asia (China), North America (U.S), South Asia (India), the Middle East (UAE), and Southern-Central Europe (Italy). The results were computed and evaluated using different scales, which included (DASS-21) Depression Anxiety Stress Scale 21, (PHQ-9) Patient Health Questionnaire 9, and (GAD-7) Generalized Anxiety Disorder 7. These scales help evaluate the data by dividing it into five levels: normal, mild, moderate, severe, and extremely severe^84^.

## Discussion

The COVID-19 pandemic has severely impacted us, whether physically, emotionally, or economically. Evidence has shown that mental health is severely underestimated or neglected. The present scenario provides an immediate need and concern to focus on the mental health condition of front-line healthcare workers assisting infected patients, and highlights the need to understand the effect on different sections of society. Early detection techniques and understanding of the necessity of treating society’s mental health conditions and coping with their psychological results must be employed. Many front-line healthcare workers, including physicians, nurses, pharmacists, and paramedical staff, have been severely impacted. Another critical aspect of the pandemic is that high psychological conditions were elevated throughout the society during the pandemic. A pandemic causes a restriction on staying in homes, which is one of the major reasons for the emergence of loneliness and depressive and anxious symptoms. Fear among workers working with COVID-19 patients generates a sense of trauma for their family members and loved ones. Economic incapability during the pandemic causes fear about society’s ability to cope with daily needs and requirements. COVID-19 has devastating effects on all areas and has affected the success of many areas. It presents an alarming need to focus on the most vulnerable groups affected by the pandemic drastically to overcome the time of crisis and secure resilient outcomes and a planned approach that reduces the risk factors for future emergencies and promotes health for all.

## Conclusion

The COVID-19 pandemic has had a huge psychological impact on healthcare workers and individuals worldwide. Individuals worldwide were studied and their mental health conditions were evaluated. People are at high risk of developing long-term psychological stress disorders or mental illnesses. Front-line healthcare workers working hard and suffering from immense workloads to help overcome the crisis and assist COVID-19-infected patients were severely affected. Families associated with them also suffered from a severe fear of being infected with COVID-19. This highlights the emerging need to provide appropriate psychological support to maintain the sustainability of healthcare services and ensure the safety and proper treatment provided to patients. Restrictive measures present an alarming need to monitor the increasing mental stress in society. Pregnant women visiting healthcare centres suffer from the fear of being infected with viruses. According to International Labor Organization (ILO) in 2020, almost 114 million peoples lost their jobs worldwide, resulting in a developing fear of economic incapability in society. Researchers conducting long-term and non-COVID-19 research studies must be postponed and cancelled. More attention must be paid to supporting the immediate crisis and providing appropriate counselling and motivation, which can help lower stress levels. Support programs can help strengthen society and encourage people to think positively, which can help lower their fear levels. Various measures and steps must be taken to reduce the intensity and workload of work to overcome future crises.

## Ethical approval

Ethics approval was not required for this review.

## Consent

Informed consent was not required for this review. All authors agreed to give consent to publish this paper.

## Source of funding

The authors declare that no funds, grants, or other support was received during the preparation of this manuscript.

## Author contribution

Conceptualization, data collection and analysis: S.C., S.A., S.M.; Literature search: M.R., H.S., A.B., M.S.A.; Manuscript writing: R.C., M.S., R.C. All authors read and approved the final manuscript and agreed to be personally accountable for the authors’ contributions and questions related to the integrity of the work.

## Conflicts of interest disclosure

The authors declare no conflict of interest in this work.

## Research registration unique identifying number (UIN)

Not applicable.

## Guarantor

Mr. Sachin Mehta, Dr. Samrat Chauhan, Dr. Shahbaz Aman.

## Data availability statement

The authors declare that data supporting the findings of this study are available in this article. The authors declare that the materials supporting the findings of this study are available in this article.

## Provenance and peer review

Not applicable.
